# The gender gap in political psychology

**DOI:** 10.3389/fpsyg.2022.1072494

**Published:** 2022-12-13

**Authors:** Jan-Erik Lönnqvist

**Affiliations:** Swedish School of Social Science, University of Helsinki, Helsinki, Finland

**Keywords:** gender gap, research profession, political psychology, authorship, gender differences

## Abstract

**Introduction:**

I investigated the authorship gender gap in research on political psychology.

**Methods:**

The material comprises 1,166 articles published in the field’s flagship journal *Political Psychology* between 1997 and 2021. These were rated for author gender, methodology, purpose, and topic.

**Results:**

Women were underrepresented as authors (37.1% women), single authors (33.5% women), and lead authors (35.1% women). There were disproportionately many women lead authors in papers employing interviews or qualitative methodology, and in research with an applied purpose (these were all less cited). In contrast, men were overrepresented as authors of papers employing quantitative methods. Regarding topics, women were overrepresented as authors on Gender, Identity, Culture and Language, and Religion, and men were overrepresented as authors on Neuroscience and Evolutionary Psychology.

**Discussion:**

The (denigrated) methods, purposes, and topics of women doing research on politics correspond to the (denigrated) “feminine style” of women doing politics grounding knowledge in the concrete, lived reality of others; listening and giving voice to marginalized groups’ subjective experiences; and yielding power to get things done for others.

## Introduction

Women are not equally represented in science in terms of publications and impact. This is especially true in male-dominated fields such as science, technology, engineering, and mathematics (STEM), in which women are severely underrepresented both in terms of raw numbers and in terms of prestige (Larivière et al., 2013; West et al., 2013). Moreover, a large authorship gender gap in publishing can be found not only in fields dominated by men but also in fields that approach or have reached gender parity in participation, such as education or psychology (West et al., 2013).

Psychology is a discipline that is often lauded by researchers who study gender gaps in academia due to the high rate of women participating in the field, from undergraduates to tenure-track professors (e.g., Ceci et al., 2014). An increasing number of women with master’s degrees and doctorates have transformed psychology from a men-dominated field to a women-dominated field. Despite this representation in numbers, women in psychology remain underrepresented as authors and in eminence—women in psychology are underrepresented in first-author publications in top journals (Brown and Goh, 2016), in the citations their work receives (Odic and Wojcik, 2020), in award received by divisions (Eagly and Riger, 2014; Brown and Goh, 2016), and in eminence (Eagly and Miller, 2016). Although political science is strongly dominated by men (Tolleson-Rinehart and Carroll, 2006; Breuning and Sanders, 2007; Teele and Thelen, 2017; Thelwall et al., 2019), this may be less true in the subfield of political psychology.

This research aimed to assess whether there is an authorship gender gap in articles published in *Political Psychology*. The journal *Political Psychology* incorporates contributions with a variety of methodologies and topics, as well as both theoretical research and applied research. Exploring how the authorship gender gap varies between different types of contributions could reveal how women are situated within the field. First off, the size of the authorship gender gap may vary as a function of method—women generally tend to employ more qualitative research methods than men (Eagly and Riger, 2014; Thelwall et al., 2019). For instance, in political science, journals that publish qualitative research tend to have more female authors than journals that publish only quantitative research (Teele and Thelen, 2017). Second, the purpose of the paper may be associated with the size of the authorship gender gap. Large-scale bibliometric analyses across all fields of research show that theoretical papers are more typical of men, whereas women are overrepresented as authors in papers intended to have a social impact (Thelwall et al., 2019). Third, authorship gender gaps may vary across topics. For instance, gender studies are one of the few social sciences fields dominated by women (Kretschmer et al., 2012), suggesting that women might not be underrepresented as authors of research on this topic.

The purpose of the present research was to investigate the possible gender gap within the field of political psychology or, more specifically, the field’s flagship journal *Political Psychology*. I expected women to be generally underrepresented as authors, but the size of the authorship gender gap varies according to the methods, purposes, and topics of the paper. Moreover, I also investigated whether the authorship gender gap has narrowed in the last decades (as suggested by Brown and Goh, 2016) and whether papers authored by women are cited less (as suggested by Odic and Wojcik, 2020). Pertinent to the last point, I also explored whether the number of citations varies with method, purpose, or topic. Some previous studies, run on articles published in the journal *Leadership Quarterly*, suggest that quantitative, review, methods, and theory articles may be cited more than qualitative articles (Antonakis et al., 2014). Women could thus be expected to be cited less, and this could at least in part be explained by different citation rates for different types of papers.

## Materials and methods

Two doctoral students, both of whom identified as women, were employed as research assistants, and they rated all articles that appeared in the journal *Political Psychology* from the start of 1997 to the end of 2021. No power calculations were made—we sought to include all articles that were available online at the time the data were collected. Between the beginning of 1997 and the end of 2021, the journal published 1,166 research articles, which constituted the material for the present study.

All articles were rated by both raters for author gender, method, purpose, and topic. Each article was rated dichotomously on all employed variables, i.e., either employing a certain method vs. not doing so, having vs. not having applied relevance, or dealing vs. not dealing with a certain topic. The rating scheme and rating criteria were discussed, developed, and revised together in the initial stages of rating, and the final rating scheme that was employed for all articles was arrived at through joint discussion after both research assistants had rated around a hundred articles. After these initial discussions, the research assistants did not discuss their ratings with each other.

The coding of methods was rather straightforward, either a given method was employed or it was not. For the article to be coded as having applied relevance, a real-world problem had to motivate a research question, or the applied relevance had to be mentioned in the abstract. Regarding the topic, the focus was on the framework within which the research was conducted. For instance, speculating upon an “identity” or “evolutionary psychology” explanation of the results did not suffice for the paper to be coded as being within these fields. In addition to discussions with the two research assistants, I relied on political psychology handbooks and research on the submissions to the journal *Political Psychology* (Mintz and Mograbi, 2015) in developing the rating scheme.

## Results

There were generally more men (*n* = 1681) than women (*n* = 991) as authors (37.1% women). Looking at the first author or single author, there were again more men (*n* = 757) than women (*n* = 409; 35.1% women), and the same was true when looking only at single authors (250 men, 126 women; 33.5% women). Women were thus similarly underrepresented both as co-authors and as lead authors.

**FIGURE 1:**
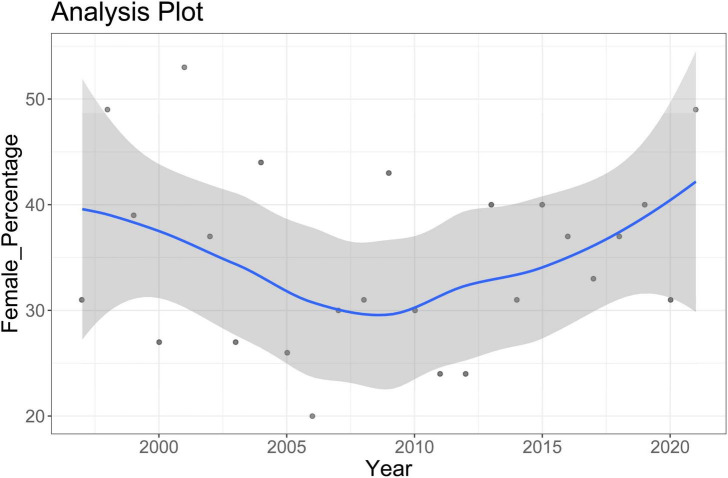
The percentage of female first authors over time.

The gender gap in lead authorship over time is plotted in Figure 1. There is no indication that the gender gap in authorship would have decreased over time [the linear correlation between the year of publication and the percentage of female lead authors was *r*(25) = 0.02]. However, the slight dip in the percentage of female lead authors suggested investigating gender representation among the editors of the journal. Between 1997 and 2021, there were two to four (co-)editors each year. The editors were all male from the beginning of 2006 to the end of 2011. All other years, there was one woman among the (co-)editors. Between 2006 and 2011, the all-male years, only 29.7% of lead authors were women. In contrast, the average of those years in which one woman was included as (co-)editor, the percentage of women lead authors was 36.6% [this difference in percentages did not reach conventional levels of statistical significance, *F*(1, 24) = 3.25, *p* = 0.084].

To analyze differences between topics, methods, and purposes, one rater’s rating of the gender of the lead author (first author or single author) was used as the independent variable (rater’s agreement on author gender was 99.5%, and kappa was 0.989).

Tables 1–3 show the number of male and female lead authors by method, purpose, and topic, respectively. Not surprisingly, given that the proportion of female lead authors was only 0.351, there were fewer women than men in almost all categories. However, there were some exceptions. Regarding methods, there were as many women as men employing qualitative methods and interviews (the number of women and men lead authors employing these methods did not differ statistically significantly at *p* < 0.05). Regarding the topic, there were as many women as men lead authors writing about Gender, Religion, Social Control, and Class (the number of women and men lead authors writing on these topics did not differ statistically significantly at *p* < 0.05).

**TABLE 1:** Number of papers with male and female lead authors across methods.

	First author					Inter-rater reliability	
	Male	Female	χ ^2^(1, *N* = 1066)	φ	*p*		
Method						% agreement	Kappa
Qualitative	124	107	15.990	0.117	< 0.001	98.714	0.959
Quantitative	570	273	10.116	–0.093	0.002	98.625	0.966
Interviews	38	53	23.257	0.141	< 0.001	99.571	0.970
Methodological	85	33	2.915	–0.050	0.103	98.971	0.944
Literature review	151	81	1.000	0.002	1.000	99.657	0.973
Meta-analysis	57	23	1.510	0.036	0.274	98.971	0.968

The Chi-square and the Phi statistics show whether either gender is disproportionately represented as lead author. The inter-rater agreement and Cohen’s kappa statistics estimate inter-rater reliability.

**TABLE 2:** Number of papers with male and female lead authors across purpose.

	First author					Inter-rater reliability	
	Male	Female	χ ^2^(1, *N* = 1066)	φ	*p*		
**Purpose**							
Solve a real world problem	143	99	4.561	0.063	0.034	97.256	0.915
Application mentioned in abstract	215	140	4.256	0.060	0.045	95.969	0.907

The Chi-square and the Phi statistics show whether either gender is disproportionately represented as lead author. The inter-rater agreement and Cohen’s kappa statistics estimate inter-rater reliability.

**TABLE 3:** Number of papers with male and female lead authors across theme.

	First author					Inter-rater reliability	
	Male	Female	χ ^2^(1, *N* = 1066)	φ	*p*		
**Theme**							
Gender	40	46	13.620	1.090	< 0.001	99.571	0.969
Identity	237	173	13.951	0.109	< 0.001	94.592	0.881
Culture and language	161	119	8.915	0.087	0.003	95.197	0.870
Religion	38	33	4.316	0.056	0.041	99.228	0.932
Political participation	126	86	3.390	0.061	0.068	95.107	0.848
Ethnicity	112	76	2.815	0.049	0.096	98.285	0.935
Social control	22	18	1.791	0.039	0.182	98.971	0.841
Voting	77	51	1.416	0.035	0.234	98.884	0.942
Class	44	31	1.378	0.034	0.261	98.971	0.916
Ideology	116	54	0.959	0.029	0.340	97.341	0.892
Leadership	58	26	0.676	0.024	0.477	99.313	0.948
Political candidates	94	57	0.543	0.022	0.466	98.885	0.951
Prejudice	181	102	0.171	0.012	0.720	96.910	0.915
International relations	48	24	0.102	0.009	0.800	99.057	0.920
Conflict management	135	74	0.012	0.003	0.936	97.770	0.925
Polarization	145	79	0.004	0.002	0.938	96.141	0.880
Values	79	43	0.002	0.001	1.000	96.821	0.824
Emotions	115	62	0.001	–0.001	1.000	97.082	0.885
Moral psychology	258	135	0.137	–0.011	0.746	93.396	0.859
Personality	200	104	0.136	–0.011	0.727	93.997	0.842
Intelligence	98	47	0.516	–0.021	0.516	96.938	0.825
Information processing	199	94	1.542	–0.036	0.229	95.455	0.882
Neuroscience	22	4	4.528	–0.062	0.037	99.828	0.959
Evolutionary psychology	25	5	4.583	–0.063	0.033	99.914	0.983

The Chi-square and the Phi statistics show whether either gender is disproportionately represented as lead author. The inter-rater agreement and Cohen’s kappa statistics estimate inter-rater reliability.

Given the generally large authorship gender gap in favor of men, more informative than analyzing the absolute numbers of men and women as lead authors were to look at the relative number of men and women. Chi-square tests were run to investigate, given the general overrepresentation of men, whether the authorship gender gap varied across methods (Table 1), purposes (Table 2), and topics (Table 3). Regarding methods, there were disproportionately many women lead authors in papers employing qualitative methods or interviews. In contrast, men were overrepresented as authors of papers employing quantitative methods. There were also disproportionately many women lead authors in research with an applied purpose. Regarding the topic, there were disproportionately many women lead authors within the fields of Gender, Identity, Culture and Language, and Religion. There were some indications that this might also be true for Ethnicity and Political Participation, although conventional levels of statistical significance were not reached. On the contrary, there were disproportionately many men as lead authors in papers on Evolutionary Psychology and Neuroscience.

To alleviate fears that the above results were being driven by only some variables, I analyzed the associations between variables: methods, purposes, and topics. The strongest association was between qualitative methods and the topic of Culture and Language, with an effect size of φ = 0.252 [χ^2^(1, 1066) = 74.784]. Entering these two variables into a binary logistic regression predicting author gender gave very similar coefficient estimates regardless of whether the two variables were entered alone or simultaneously (all coefficient *p*-values in both types of models were statistically significant at *p* < 0.01). This suggests that the associations between, on the one hand, methods, purposes, and topic and, on the other hand, gender were generally rather independent of each other.

Regarding the number of citations, there was no gender bias. Male lead authors had an average of 102 (*SD* = 192) citations, and female first authors had an average of 93 [*SD* = 166; *F*(1, 1164) = 0.722, ns]. Of the 50 most-cited articles, 18 (36%) had a female lead author, which is almost exactly what one would expect given that 37% of all lead authors were women. Similarly, when looking at the top 10 most-cited articles, three of them had a female lead author.

Although articles authored by women were, contrary to expectations, not cited less frequently, the types of papers for which women were overrepresented as authors were cited less frequently. As expected, papers employing qualitative methods (Table 4) and having an applied focus (Table 5) were cited less often. However, the topics that women authors were overrepresented on were not cited particularly poorly, with the exception of Culture and Language (Table 6).

**TABLE 4:** Mean number of citations across employed methods.

	Method employed		Method not employed				
	Number of articles	Mean number of citations (SD)	Number of articles	Mean number of citations (SD)	*F*(1, 1164)	η ^2^	*p*
**Method**							
Qualitative	227	60.17 (99.88)	939	108.40 (197.15)	12.785	0.011	< 0.001
Quantitative	845	96.33 (141.84)	319	105.89 (263.82)	0.627	0.001	0.429
Interviews	92	46.64 (57.30)	1074	103.50 (189.52)	8.204	0.007	0.004
Methodological	120	170.88 (364.99)	1046	90.77 (147.04)	20.916	0.18	< 0.001
Literature review	244	137.19 (303.64)	922	88.91 (132.97)	13.532	0.011	< 0.001
Meta-analysis	80	256.36 (456.29)	1086	87.42 (137.67)	66.885	0.054	< 0.001

*F* and η^2^ statistics for the difference in mean citations between papers in which the method was or was not present.

**TABLE 5:** Mean number of citations across purposes.

	Purpose present		Purpose not present				
	Number of articles	Mean number of citations (SD)	Number of articles	Mean number of citations (SD)	*F*(1, 1164)	η ^2^	*p*
**Purpose**							
Solve a real-world problem	230	73.77 (136.74)	936	105.21 (192.59)	5.454	0.005	0.020
Application mentioned in abstract	378	64.30 (126.96)	789	115.66 (292.86)	20.397	0.017	< 0.001

*F* and η^2^ statistics for the difference in mean citations between papers in which the purpose was or was not present.

**TABLE 6:** Mean number of citations across themes.

	Theme present		Theme not present				
	Number of articles	Mean number of citations (SD)	Number of articles	Mean number of citations (SD)	*F*(1, 1164)	η ^2^	*p*
**Theme**							
Gender	89	101.16 (189.06)	1077	72.99 (83.52)	1.944	0.002	0.164
Identity	407	93.82 (226.90)	758	101.91 (155.10)	0.515	0.000	0.473
Culture and language	290	73.78 (120.87)	876	107.36 (199.06)	7.357	0.006	0.007
Religion	71	103.86 (200.21)	1095	98.70 (182.23)	0.053	0.000	0.818
Political participation	257	83.17 (130.09)	908	103.39 (195.64)	2.439	0.002	0.119
Ethnicity	178	65.50 (80.62)	988	105.05 (195.57)	7.058	0.006	0.008
Social control	38	43.18 (86.75)	1128	100.89 (185.40)	3.652	0.003	0.056
Voting	123	100.45 (146.40)	1042	98.43 (186.80)	0.13	0.000	0.908
Class	77	71.03 (100.21)	1089	100.99 (187.65)	1.923	0.002	0.166
Ideology	165	145.45 (324.78)	1001	91.35 (146.37)	12.462	0.011	< 0.001
Leadership	82	62.06 (73.48)	1083	101.89 (189.81)	3.603	0.003	0.058
Political candidates	154	75.03 (84.68)	1012	102.86 (193.72)	3.042	0.003	0.081
Prejudice	275	87.01 (233.58)	891	102.71 (164.68)	1.544	0.001	0.214
International relations	75	59.16 (74.32)	1091	101.75 (188.19)	3.798	0.003	0.052
Conflict management	211	84.79 (135.06)	954	102.26 (192.31)	0.922	0.002	0.398
Polarization	247	79.85 (134.28)	919	104.16 (194.10)	3.433	0.003	0.064
Values	112	118.10 (201.02)	1053	97.04 (181.37)	1.336	0.001	0.248
Emotions	171	75.94 (104.84)	994	103 (195.45)	3.184	0.003	0.075
Moral psychology	468	84.97 (124.05)	698	108.42 (213.59)	4.599	0.004	0.032
Personality	292	91.40 (179.65)	874	101.55 (184.52)	0.672	0.001	0.413
Intelligence	127	85.50 (104.83)	1039	100.66 (190.65)	0.775	0.001	0.379
Information processing	316	76.13 (118.39)	850	107.52 (201.57)	6.787	0.006	0.009
Neuroscience	25	66.60 (69.29)	1141	99.72 (184.95)	0.799	0.001	0.372
Evolutionary psychology	29	54.59 (57.79)	1137	100.14 (185.25)	1.748	0.001	0.186

*F* and η^2^ statistics for the difference in mean citations between papers in which the theme was or was not present.

## Discussion

The first finding was that only 37.1% of the authors who published their articles in *Political Psychology* in 1997–2021 were women (33.5% single authors, 35.1% first authors). The average for the top 200 psychology journals from 2003 to 2018 was 44.2% (Odic and Wojcik, 2020). The most important finding for a wider audience was that the authorship gender gap was not identical across various methodological approaches, research purposes, and research topics—i.e., women scholars were different in how (methods), why (purpose), and what (theme) they did. This result was especially intriguing given that the journal investigates politics. Politics is of course not gender-neutral—women politicians are expected to be different in how, why, and what they do. One of the questions the results provoke is whether research on politics is gendered similarly to how politics is gendered. That is, gender differences in what political psychologist do may be parallel to gender differences in what politicians do.

Regarding methodology, women were, relative to the general authorship gender gap in favor of men, overrepresented as lead authors in research employing qualitative methods and interviews. Women were also overrepresented in research with an applied purpose. These results are consistent with previous large-scale cross-disciplinary bibliometric results (Thelwall et al., 2019). The similarities between how and why women do research and how and why women do politics are striking. A “feminine style” in women’s political discourse has been argued to consist, among other things, of basing political judgments on concrete, lived experience; valuing inclusivity and the relational nature of being; conceptualizing the power of public office as a capacity to “get things done”; and moving women’s issues to the forefront of the public arena (Blankenship and Robson, 1995). This “feminine style” ascribed to women politicians could just as well describe the results for women scholars. Several approaches within qualitative research, especially when interviews are employed, have been thought of as seeking to capture “lived experience” (Frechette et al., 2020), legitimize the subjectivity of human reality (Morgan and Drury, 2003), or “give voice” to those who are rarely heard (Larkin et al., 2006). Women in politics and women scholars seem to ground their knowledge in the concrete, lived reality of others (their electorate or their research participants), listening and giving voice to marginalized groups’ subjective experiences, and yielding their own power or their voice for the sake of others. This is all very consistent with the broader stereotype of women as highly communal (i.e., kind, warm, empathetic, and caring) and less agentic (i.e., analytical, independent, and competitive) than men (Fiske et al., 2002).

In addition, consistent with these broader stereotypes are the topics, on which women were, given the general authorship gender gap in favor of men, overrepresented: Gender, Identity, Culture and Language, and Religion. Two topics that narrowly missed the cut-off points for statistical significance were Political participation and Ethnicity. It is again rather striking that the topics women scholars work on are the very same type of topics that women politicians are associated with. In electoral politics, even though women’s overall proportions in the governments of developed democracies have increased considerably over the last few decades, women cabinet ministers in charge of the most prestigious (i.e., pivotal, resourceful, and visible) positions remain an exception (Krook and O’Brien, 2012; Kroeber and Hüffelmann, 2021). Women members of government typically preside over low-prestige portfolios, such as Women, Equality, Minority Affairs, Culture, Minority Affairs, and Immigration (Kroeber and Hüffelmann, 2021). These portfolios are very similar in content to the above topics on which women authors were overrepresented.

Besides affinities in the methods, purposes, and topics, of women scholars and politicians, there are similarities in the prestige that they are afforded. Regarding research methods, policymakers are infatuated with the number and quantitative methods, particularly at the intersection of science and bureaucracy (Porter, 1995). Also in the present study, qualitative research was poorly cited. This was consistent with previous research on the citation rates of leadership research (Antonakis et al., 2014), as well as with the present result that papers with a more applied focus were poorly cited. That applied papers, like qualitative papers, may lack prestige can be inferred not only from citation rates but also from a recent Association for Psychological Science presidential column, in which it was noted that “Many of us in academia may be walking around with an implicit or explicit ‘basic is better’ attitude” (Medin, 2012). Thus, although women were not *per se* less likely to be cited than men, the type of research that women did was less likely to be cited.

Regarding the topics of the research, Gender, Identity, Culture and Language, and Religion, also maybe Ethnicity and Political Identity, are all topics that in eyes of both other psychologists and laypeople are perceived as less rigorous, mainstream, and objective than research in areas of psychology, and the researchers engaged with this type of research on identity or “me-search” (Rios and Roth, 2020) are seen as more subjective and less intelligent (Rios and Roth, 2020; Brown et al., 2022).

Both women politicians and scholars appear to focus on similar issues, and these issues are denigrated. In retrospect, this is perhaps not that surprising. Just as “intelligence” in political leadership is associated with masculinity (for a meta-analysis, see Koenig et al., 2011), so is being a “rational” and an “objective” scientist (Carli et al., 2016). These parallels offer intriguing questions for future research. For instance—given that female politicians can increase their chances of electoral success by focusing on “soft” issues in the election campaign (Herrnson et al., 2003)—one can ask whether papers authored by women are more likely to be accepted if they focus on “soft” topics, are qualitative, or are applied. Another instance, as in politics, in which men can capture “soft issues” (Herrnson et al., 2003), are qualitative papers or papers on “women’s topics” more likely to be published when authored by men? On a very general level, it would be interesting to investigate whether “women’s topics” are considered less scientific because women focus on these topics, or whether women focus on these topics because they are rejected when venturing into areas perceived as requiring more masculine characteristics, such as “rationality” and “objectivity” (Carli et al., 2016).

Those men were overrepresented in research employing quantitative methods and in research rooted in Neuroscience and Evolutionary Psychology also aligns well with the above-discussed gender stereotypes. Although the number of papers on these topics was rather small, the notion that at least Evolutionary Psychology (e.g., Meredith, 2013) is dominated by men aligns well with previous research. In terms of future research, our results regarding the overrepresentation of men do offer some intriguing suggestions. Both Neuroscience and Evolutionary Psychology can be argued to take a rather essentialist approach. For instance, in a much-cited paper, DeLamater and Hyde (1998) argued that modern essentialism, within which they count both Evolutionary Psychology and Brain Research, consists of a belief that certain phenomena are natural, inevitable, and biologically determined. They contrast this belief with that of social constructionism, the belief that reality is socially constructed, which emphasizes language as an important means by which we interpret experience. Essentialist claims about inevitable differences with regard to sex and gender have been argued to be incompatible with feminism (e.g., Kelly, 2014), and the controversies surrounding this topic have come to reach larger audiences (e.g., Hufendiek, 2022). This could in part explain why women do not venture into working on these themes. Interesting questions could be whether men are disproportionately overrepresented as authors in other psychological or social sciences research that takes an essentialist approach to sex or more broadly an essentialist approach to individual differences.

An important takeaway for future research is that merely looking at the numerical representation of women is not enough. Probing deeper by looking at authorship gender gaps as a function of the topics, methods, and purposes of the research can shed light on the processes underlying these gaps. To what extent can research also within other disciplines be described as women researching (possibly derided) women’s issues and, in doing so, employing certain (possibly derided) methods?

## Data availability statement

The raw data supporting the conclusions of this article will be made available by the authors, without undue reservation.

## Author contributions

The author confirms being the sole contributor of this work and has approved it for publication.
